# Evaluating the Usability, Durability, and Effectiveness of Permethrin-Treated Uniforms and Metofluthrin Spatial Repellent in Preventing Mosquito Bites in the Laos Military

**DOI:** 10.3390/tropicalmed11050142

**Published:** 2026-05-19

**Authors:** Parat Boonyarangka, Pheutsapha Sonthilath, Sidhartha Chaudhury, Virasack Banouvong, Worachet Kuntawunginn, Siriporn Sornsakrin, Bounor Taobouathong, Thavone Sinhthapaseuth, Utaiwan Srichairatanakul, Montri Arsanok, Chanthalone Khamkong, Latsamee Lattavong, Raweewan Srisawat, Tharinee Saleepochn, Witcha Imaram, Alyssa Mann, Erica J. Lindroth, Bouasy Hongvanthong, James Jones, Krisada Jongsakul

**Affiliations:** 1 Walter Reed Army Institute of Research, Armed Forces Research Institute of Medical Sciences, Bangkok 10400, Thailandutaiwans.fsn@afrims.org (U.S.);; 2 Laos People’s Army Medical Department, Vientiane 01000, Laos; 3 Laos Center of Malariology, Parasitology, and Entomology (CMPE), Vientiane 01000, Laos; banouvong@gmail.com (V.B.);; 4 Department of Medical Entomology, Faculty of Tropical Medicine, Mahidol University, Bangkok 10400, Thailand; raweewan.sri@mahidol.ac.th; 5 Department of Chemistry, Faculty of Science, Kasetsart University, Bangkok 10900, Thailand; 6 Center of Excellence for Innovation in Chemistry, Faculty of Science, Kasetsart University, Bangkok 10900, Thailand

**Keywords:** malaria, military, vector control, permethrin, metofluthrin

## Abstract

While vector control methods have successfully reduced malaria rates worldwide, such methods are not always suitable for military use. We evaluated the usability, durability, and effectiveness of permethrin-treated uniforms and metofluthrin spatial repellent in preventing mosquito bites among soldiers in the Laos People’s Army that were deployed on a 12-week field exercise. The study enrolled 173 subjects into four different groups: (1) metofluthrin-only, (2) permethrin-treated uniforms, (3) metofluthrin with permethrin-treated uniforms, and (4) a control group. We measured efficacy through self-reported survey responses and a serological test for antibodies to *Anopheles* salivary antigens and assessed the durability of permethrin treatment over the 12-week exercise. We found that soldiers given metofluthrin and permethrin-treated uniforms were 72% and 46% more likely to report decreased mosquito bites compared to those in the control group and that soldiers with permethrin-treated uniforms had significantly lower (*p* < 0.05) antibody levels to the *Anopheles* SG6 salivary antigen. Mass spectrometry analysis revealed that treated uniforms still contained a significant permethrin concentration that showed 50 to 100% mosquito mortality. Overall, our findings show that both metofluthrin and permethrin-treated uniforms were well-tolerated and led to significantly fewer reported mosquito bites, and that permethrin, in particular, was found to be durable throughout the 12-week field exercise and effective in reducing mosquito bite exposure, underscoring its value in military applications.

## 1. Introduction

Mosquito-borne infectious diseases such as malaria and dengue fever remain a persistent threat to military service members deployed to endemic regions in Asia, Africa, and South America. Vector control measures such as bed nets and repellents have long been demonstrated to be effective in reducing malaria rates in public health settings and form the cornerstone of malaria control and elimination efforts. However, these control measures are often not suitable for military personnel deployed to a field environment for extended periods of time, where they typically reside in temporary shelters and bed nets or regular access to insect repellents may not be feasible. Identifying effective vector control measures in military settings is critical to prevent outbreaks of malaria among deployed personnel.

Despite the advent of numerous effective chemoprophylaxis regimens in the 20th and 21st centuries, including doxycycline, mefloquine, and atovaquone–proguanil (Malarone™), peacekeeping and other military operations continue to be impacted by malaria during operations in endemic regions. In 2006, a French unit of 575 personnel returning from a 4-month mission to Côte d’Ivoire experienced 39 cases of malaria including three serious cases mainly due to *P. falciparum* malaria, despite being given doxycycline as chemoprophylaxis [1]. In 2003, 44 U.S. marines out of a unit of 225 personnel that were deployed to Liberia in response to civil unrest needed to be evacuated due to malaria [2] while taking mefloquine chemoprophylaxis. Mefloquine prophylaxis failure was also reported by Guatemalan soldiers deployed to the Democratic Republic of Congo in 2010 [3,4] and Peruvian soldiers deployed to the Central African Republic in 2016–2022 [5,6] for UN Peacekeeping Missions. In 2023, a Royal Thai Army unit of 273 soldiers under mefloquine prophylaxis experienced 64 cases of malaria during a single 12-month UN Peacekeeping Mission deployment to South Sudan [7]. These findings highlight the limitations of relying solely on chemoprophylaxis and underscore the importance of vector control measures in protecting military personnel from malaria during these missions.

Vector control measures, such as insecticide-treated nets (ITNs) and indoor residual spraying (IRS), have historically played a critical role in reducing malaria disease burden [8]. However, the efficacy of vector interventions suitable for militaries, including the use of insecticide-impregnated uniforms and various forms of personal and spatial repellents, remains relatively untested in field settings. Permethrin is the most common insecticide used for treating clothing. Prior studies evaluating permethrin-treated uniforms in preventing malaria did find statistically significant reductions in malaria in both civilian [9] and military populations [10], with a duration of efficacy estimated to be at least 2–3 months. These studies were conducted in the absence of chemoprophylaxis and suggested that insecticide-treated uniforms could play a significant role in malaria prevention. Further studies on the efficacy of insecticide-treated uniforms and topical repellents on malaria infection rates in high-transmission areas are on-going [11]. The use of insecticide-treated uniforms is widespread in many Western militaries when deploying to regions with a high prevalence for arthropod-borne diseases, where these measures have been shown to have high efficacy [12,13,14]. In Asian countries, however, vector control is usually limited to repellent lotions, topical sprays and ITNs, and does not typically extend to treated uniforms.

The present study had four objectives. First, to evaluate the effectiveness of metofluthrin spatial repellent and permethrin-treated uniforms in reducing the frequency of mosquito bites. Second, to assess the usability and acceptability of these two interventions by soldiers for use in a military field environment. Third, to evaluate permethrin-treated uniforms in terms of chemical durability following 12 weeks of regular use and wear in the field in terms of the retention and activity of permethrin in the uniform fabric. Fourth, to use a new serology test to evaluate exposure to mosquito bites in the different intervention groups. To our knowledge, this study is the first to use a serological test for mosquito bite exposure in addition to survey responses on perceptions of mosquito bite frequency to assess the effectiveness of vector control measures in a military field setting.

## 2. Materials and Methods

A multi-arm study evaluating the usability, durability, and effectiveness of permethrin-treated uniforms and metofluthrin spatial repellent in reducing exposure to *Anopheles* bites was conducted by the Laos People’s Army (LPA) in Champasak Province in southern Laos in 2022 with support from the Walter Reed Army Institute of Research-Armed Forces Research Institute of Medical Sciences (WRAIR-AFRIMS). This clinical project was approved by the Institutional Review Board at Laos National Ethical Committee for Health Research (Protocol number WR2882, approved 3 February 2022). The WRAIR-AFRIMS involvement in the study was classified as Non-Human Subjects Research by the WRAIR Human Subjects Protection Branch. Subject participation in the investigation was voluntary and research staff included members from the WRAIR-AFRIMS, the Laos Center for Malariology, Parasitology, and Entomology (CMPE), and the LPA, as well as the Champasak Health Office and Champasak Military Health Office.

### 2.1. Study Population

The sample population consisted of military units participating in a field exercise conducted in conjunction with this project. Eligible participants were adult military personnel serving in these units. Inclusion criteria were as follows: (1) LPA soldiers; (2) deployment to LPA sub-camps in Champasak Province during the study period; (3) age between 18 and 60 years prior to survey initiation; and (4) provision of written informed consent. A total of 177 participants who met the eligibility criteria were enrolled in the investigation.

Four military units (cohorts) participated in the study, and each was assigned a different intervention condition. All personnel were issued their standard equipment and material for the field exercise. Cohort 1 was given metofluthrin spatial repellent as the primary form of vector control. Cohort 2 was given permethrin-treated uniforms along with DEET insect repellent. Cohort 3 was given both the interventions of Cohort 1 and Cohort 2—metofluthrin repellent with treated uniforms and DEET. Cohort 4 was the control group and was not given any specific vector control method. These cohorts were assigned to different locations during the field exercise, within the same geographical area in Champasak Province, along the Mekong River. The research was unblinded with respect to intervention conditions.

### 2.2. Insectacide-Treated Uniforms and Spatial Repellent

For Cohorts 2 and 3, uniforms were treated with permethrin by spraying them with a permethrin formulation using a two-gallon sprayer, as described in [15]. A team from the AFRIMS was sent to the study site to train LPA personnel on permethrin uniform treatment and to oversee the uniform treatment process. All soldiers in the study received new uniforms at the start of the study, regardless of whether they were selected for uniform treatment. Permethrin insecticide was purchased from Tracy Defense Depot USARMY (Tracy, CA, USA). Metofluthrin was provided using the MozzieMesh™ spatial repellent product (Sumitomo Chemicals, Bangkok, Thailand). MozzieMesh™ is a small disposable metofluthrin-impregnated mesh product that is typically hung at a fixed location and passively emits the repellent through evaporation without the use of heat or electricity. According to the manufacturer, MozzieMesh™ provides continuous repellency for up to 30 days after opening. In this study, soldiers were asked to place the product in a vented mesh pouch attached to their uniform belt and given a replacement every three weeks during the study. DEET was provided using Defense Repellent Milky Lotion 33% (weight per weight) 50 mL product, produced by Defense Pharmaceutical Factory (Bangkok, Thailand).

### 2.3. Surveys

Surveys were conducted at enrollment and at the conclusion of the field exercise at week 12 and asked questions about demographics, medical history, awareness of malaria risks, and preferred vector control measures. At week 12, subjects were assessed in terms of feedback on the side effects of the vector control measures, perceptions of mosquito bite frequency, time of day of mosquito biting, and whether they felt their vector control interventions had any effect on the number of mosquitos around them and the number of mosquito bites they experienced compared to their prior experience.

### 2.4. Serological Assay

We assessed antibody responses to *Anopheles* mosquito salivary SG6 antigen for *Anopheles dirus*, *Anopheles minimus*, and *Anopheles maculatus* using the MesoScale U-Plex platform utilizing 10-spot antigen plates (MSD, Gaithersburg, MD, USA), performed as described previously [16,17]. Briefly, biotinylated proteins were diluted to a concentration of 300 nM using coating diluent (PBS with 0.5% BSA) and linked with a unique U-plex linker provided by the U-PLEX platform (MSD), vortexed, and incubated at room temperature (RT) for 30 min. The U-PLEX-coupled protein solutions were brought up to 6 mL with Stop Solution, creating a multiplex coating solution. Plates were coated with the cocktail of proteins and incubated at RT for 1 h on a Titramax plate shaker (Heidolph, Schwabach, Germany), shaking at 700 rpm. Coated plates can be stored for up to seven days at 2–8 °C based on the manufacturer’s protocol. After incubation, the plates were washed with a working solution of MSD Wash Buffer (MSD) three times. Sera were diluted to the desired concentration with Diluent 2 (MSD), added to each well and incubated at RT for 1 h on a plate shaker. Plates were washed three times with MSD Wash Buffer and incubated with a detection antibody, SULFO-TAG goat anti-human antibody (diluted to 1 µg/mL in Diluent 3 (MSD)). Plates were sealed and incubated at RT for 1 h on a plate shaker (700 rpm). After washing, MSD Read Buffer T was added to each well and the plates were read on the MESO QuickPlex SQ 120 reader (MSD, Gaithersburg, MD, USA), per the manufacturer’s instructions.

### 2.5. Permethrin Durability Assay

After 12 weeks of the field exercise, permethrin concentrations in treated uniforms were quantified by gas chromatography–mass spectrometry (GC-MS). Three permethrin-treated uniform samples and two untreated uniform samples were submitted to the Department of Chemistry, Faculty of Science, Kasetsart University (Bangkok, Thailand), for GC-MS analysis using a Shimadzu GCMS QP-2020 gas chromatograph–mass spectrometer (Shimadzu, Kyoto, Japan). Analytes were separated on a VF-5MS capillary column (60 m × 0.25 mm i.d., 0.25 μm film thickness; Varian, Middelburg, The Netherlands). Helium was used as the carrier gas at a constant flow rate of 1.0 mL/min. The oven temperature program was as follows: initial temperature of 150 °C, ramped to 200 °C at 20 °C/min, then increased to 250 °C at 10 °C/min, and finally increased to 280 °C at 10 °C/min. Samples (1 µL) were injected at an injector temperature of 280 °C in split mode with a split ratio of 20:1. Data were acquired in selected ion monitoring (SIM) mode. The ion source and transfer line temperatures were maintained at 230 and 280 °C, respectively. Target and qualifier ion abundances were previously determined by injection of permethrin standards under the same conditions. GC-MS analysis in SIM mode employed one target ion and three qualifier ions. Permethrins were identified based on retention times, and quantification was performed using the peak area of the target ion. Uniform samples were cut into 12 cm × 15 cm pieces, further divided into four triangular sections and placed in a 15 mL centrifuge tube for extraction. Extraction was performed using an 80/20 (*v*/*v*) acetonitrile/methanol mixture with sonication in an ultrasonic bath at RT for 20 min, followed by the addition of 1 g sodium chloride and storage at −18 °C for 1 h. The samples were then warmed to RT and centrifuged at 4500 rpm for 5 min. An aliquot of 5 mL acetonitrile was transferred to a 15 mL dispersive solid-phase extraction tube containing 150 mg primary secondary amine (PSA), 300 mg of C18, and 900 mg of anhydrous magnesium sulfate. The tube was vortex-mixed vigorously for 1 min and centrifuged at 4500 rpm for 5 min. The resulting supernatant was filtered through a 0.45 μm nylon membrane filter into an injection vial for GC-MS analysis.

### 2.6. Permethrin Activity Bioassay

We tested treated uniform samples for efficacy in terms of knockdown rate and lethality rate using the WHO cone bioassay against laboratory bred strains of *An. dirus* and *An. minimus*. We introduced 5 non-blood-fed susceptible female mosquitos aged 2–5 days into the WHO plastic cones. The mosquitos were left to rest with contact for 4 min and then gently removed using an aspirator. The mosquitos were placed in 150 mL paper cups with 5% sugar solution and maintained for 24 h at RT. We recorded knockdown mosquitos at 1 h post-exposure and mortality after 24 h. Ten replicates of five mosquitos were used for each sample tested. Results were pooled for analysis. The control sample was an untreated uniform sample.

### 2.7. Statistical Analysis

Demographic, epidemiological, and laboratory data were summarized at baseline and follow-up. T-tests and chi-square tests were used to assess the statistical significance of differences in two means (including log-transformed) or proportions, respectively. For survey responses, answers were coded as a binary response (for example “much fewer mosquito bites” vs. “not much fewer mosquito bites”) and a mixed-effects logistic regression model was used to assess the role of intervention (metofluthrin and permethrin-treated uniforms) on the binary response. Random effects were modeled at the cohort level. For the serological data, a generalized linear model was used to model the antibody level to the SG6 antigen based on three independent variables of spatial repellent usage, treated uniform usage, and time point.

## 3. Results

### 3.1. Demographics and Survey Responses

The study enrolled a total of 173 subjects across four cohorts. The participants were all adult male, in the following age ranges: 19–30 years old (50%), 31–40 years old (34%), and 41 to 60 years old (16%). Soldiers consisted of enlisted or non-commissioned officers (73%) and officers (27%). Most subjects (89%) reported completing secondary school as their highest level of education. In terms of patrol activities, 24%, 69%, and 7% of participants reported going on patrol 1–3 days a month, 3–5 days a month, and more than 5 days a month, respectively. There was no significant difference between the four cohorts in terms of their monthly frequency of patrol. We asked about differences in vector control measures used in camp compared to those used on patrol. In camp, mosquito nets were the most commonly used method (90%), followed by use of burn coils (23%) and insect repellent 5%. On patrol, the use of vector control measures was far less, with 26% using mosquito nets, 11% using repellent, and 8% using burn coils. Nearly all soldiers (95%) listed ‘repellent’ as the preferred control method of choice that they would ‘like to have’.

In terms of medical history specifically related to malaria, there was a range of medical histories. A majority of soldiers (59%) reported never having been diagnosed with malaria. Among those that did report having malaria in the past, 13%, 12%, 8%, and 9% reported having malaria once before, twice before, three times before, and more than three times before, respectively. Most soldiers that have had malaria in their lifetime had their most recent malaria episode in the past 1–5 years (47%), with 11% having had an episode in the past year, 30% having had it in the past 5–10 years, and 11% having had it more than ten years ago.

### 3.2. Perceptions About Mosquito Bites and Side Effects

**Figure 1:**
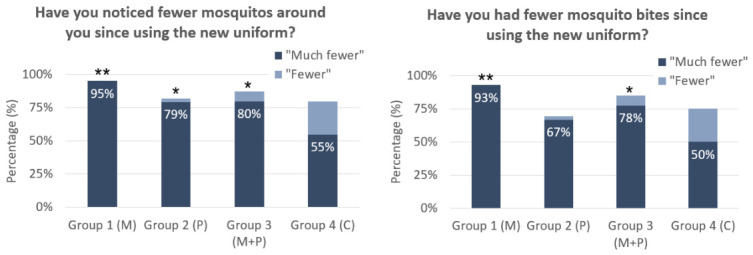
Percentage of subjects that reported ‘fewer’ (light blue) and ‘much fewer’ (dark blue) mosquitos around (**left**) and mosquito bites (**right**) for the four groups: metofluthrin (Cohort 1, M), permethrin-treated uniforms (Cohort 2, P), metofluthrin and permethrin-treated uniforms (Cohort 3, P + M), and the control group (Cohort 4, C). Statistical significance shown for *p* < 0.05 (*) and *p* < 0.01 (**) based on a logistic regression analysis.

In the week 12 survey, subjects were asked whether they had noticed fewer mosquitos around them since using the new uniform and whether they experienced fewer mosquito bites (Figure 1). In the control group, 55% of subjects reported ‘much fewer mosquitos around’ them and 50% reported ‘much fewer mosquito bites’. Still, the vector control measures did appear to make a difference. Among soldiers that were given metofluthrin repellent (Cohorts 1 and 3), 87% reported ‘much fewer mosquitos around them’ and 86% reported ‘much fewer mosquito bites’, meaning they were 58% and 72% more likely than the control group to report fewer mosquitos and fewer bites, respectively. Likewise, for soldiers given permethrin-treated uniforms (Cohorts 2 and 3), 80% reported ‘much fewer mosquitos around’ them and 73% reported ‘much fewer mosquito bites’, meaning they were 45% and 46% more likely than the control group to report fewer mosquitos and fewer bites, respectively. We carried out a logistic regression on the likelihood of reporting ‘much fewer mosquitos around’ and ‘much fewer mosquito bites’ and found that metofluthrin and permethrin were both associated with a significantly higher likelihood of reporting much fewer mosquitos around (*p* < 0.01 and *p* < 0.05, respectively) and much fewer mosquito bites (*p* < 0.01 and *p* < 0.05, respectively). These results suggested that soldiers did perceive both the metofluthrin and permethrin-treated uniforms to be effective in reducing mosquito bite exposure.

**Figure 2:**
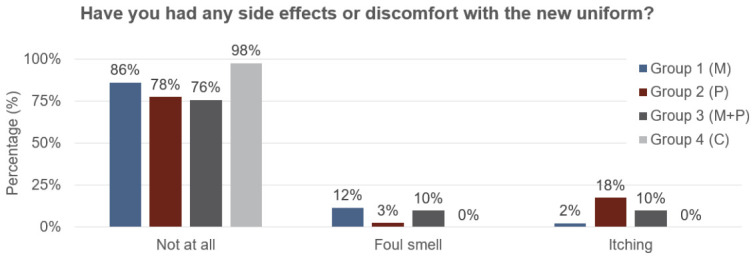
Percentage of subjects that reported side effects or discomfort associated with the vector control measures for metofluthrin (Group 1, M), permethrin-treated uniforms (Group 2, P), metofluthrin and permethrin-treated uniforms (Group 3, M + P), and the control group (Group 4, C). Subjects were specifically asked to respond with ‘not at all’, ‘foul smell’ or ‘itching’.

In the week 12 survey, we also asked subjects whether they experienced any side effects of the vector control measures, such as ‘foul smell’ or ‘itching’. Overall, we found that both metofluthrin and permethrin-treated uniforms were well tolerated, with at least 75% of subjects in all groups reporting no major side effects or discomfort, but we did find evidence of discomfort associated with the vector control measures (Figure 2). In the control group, 98% of subjects reported to not have any side effects or discomfort, compared to 86% of subjects in Cohort 1 (M), 78% in Cohort 2 (P), and 76% in Cohort 3 (P + M). We found that, overall, 11% of volunteers that received metofluthrin (Cohorts 1 and 3) reported ‘foul smell’, compared to 3% of Cohort 2 and 0% of Cohort 4. Likewise, we found that, overall, 14% of subjects that received permethrin-treated uniforms reported itching, compared to 2% in Cohort 1 and 0% in Cohort 4. These findings highlight that metofluthrin use appeared to be associated with foul smell and permethrin-treated uniforms appeared to be associated with itching.

### 3.3. Serological Exposure to Mosquito Bites

**Figure 3:**
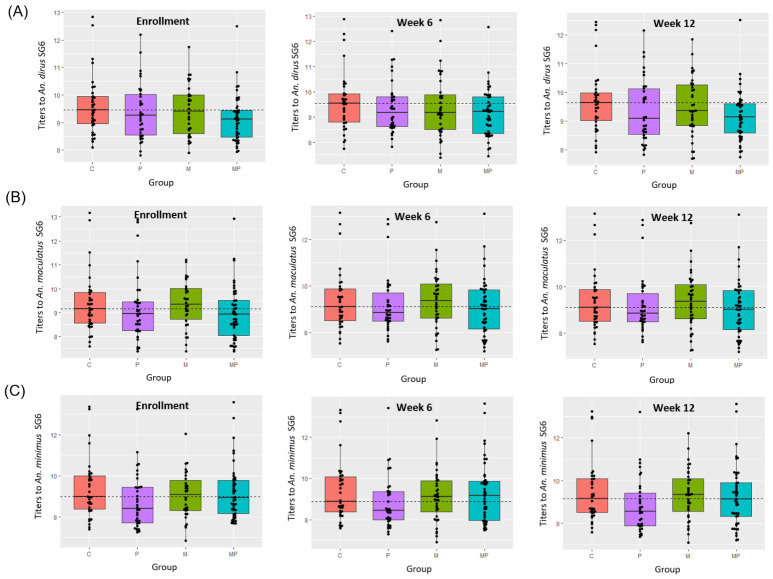
Serological assay measuring antibody response to the SG6 antigen for *An. dirus* (**A**), *An. maculatus* (**B**), and *An. minimus* (**C**) at enrollment (**left**), Week 6 (**middle**) and week 12 (**right**) for control group (red), permethrin-treated uniform group (purple), metofluthrin group (green), and the group with both permethrin-treated uniforms and metofluthrin (blue). The median antibody response at enrollment across all groups is shown as a dotted line. Antibody responses are log transformed.

We used a multiplex serological assay to measure antibody responses to *Anopheles* salivary antigen SG6 [18] from *An. dirus*, *An. minimus*, and *An. Maculatus* (Figure 3). Serum samples were collected at enrollment, Week 6 and week 12. Antibody responses were converted to log scale and then analyzed using a linear regression model that modeled the antibody response to each antigen as a function of intervention type (metofluthrin and permethrin-treated uniform) and time point. We found that at enrollment there was no significant difference in the antibody levels to *An. dirus* SG6 antigen between the four cohorts. At Week 6, there was no statistically significant effect of permethrin or metofluthrin on antibody levels to SG6, but a trend could be seen of decreasing antibody levels in the treated cohorts compared to the control cohort. By week 12, there was a significant difference, with the permethrin-treated groups (Cohorts 2 and 3) showing significantly reduced antibody levels to SG6 antigen (*p* < 0.05) relative to the control group and the metofluthrin-only group. Logistic regression showed that the use of permethrin was associated with a reduction in anybody levels to SG6 at week 12, but that metofluthrin was not. No statistically significant change in antibody responses to *An. minimus* and *An. maculatus* was observed from enrollment to Week 6 to week 12 under any of the treatment conditions.

### 3.4. Permethrin Durability and Activity

**Figure 4:**
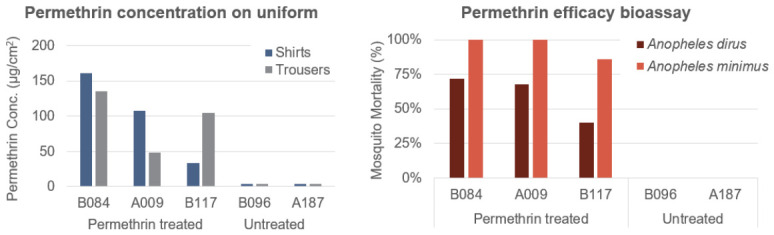
Permethrin concentration and efficacy of treated uniforms following 12 weeks of regular use and washing in the field. (**Left**) Permethrin concentration on treated (samples# B084, A009, B117) and untreated uniforms (samples# B096, A187) for shirts (blue) and trousers (gray). (**Right**) Results of mosquito mortality bioassay on mosquito mortality for the same uniform samples for laboratory-raised *An. dirus* (dark red) and *An. minimus* (orange).

We used GC/MS to measure permethrin concentration in sample uniforms after 12 weeks of the field exercise. Samples from three treated uniforms were used along with two non-treated uniforms as controls (Figure 4). GC/MS results showed that a significant concentration of permethrin remained on the uniforms after 12 weeks of regular use and washing. The average concentration in the treated uniforms was 100.6 µg/cm^2^ on samples from shirts and 96.1 µg/cm^2^ on samples from trousers, compared to a background concentration of 3.5 ug/cm^2^ from both shirts and trousers in the untreated uniforms.

**Figure 5:**
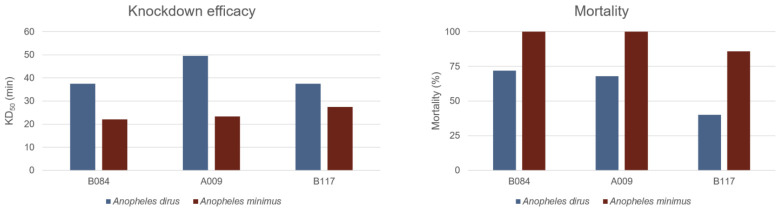
Permethrin efficacy following 12 weeks of field training. (**Left**) The estimated time when 50% of *An. dirus* and *An minimus* mosquitos are knocked down (KD_50_, minutes). (**Right**) The mortality after exposure to permethrin-treated uniform samples (# B084, A009, B117) following field use.

We used a laboratory bioassay to measure the activity of the permethrin-treated uniform samples after the field exercise by measuring both mosquito knockdown and mosquito mortality. In terms of knockdown, permethrin-treated uniform samples showed activity against two mosquito species: *An. dirus* and *An. minimus*. The median knockdown time (KD_50_) for *An. dirus* ranged from 37.49 to 49.51 min for the three uniform samples (B084, A009, and B117). For *An. minimus*, knockdown occurred faster, with KD_50_ values ranging from 22.03 to 27.44 min (Figure 5). In all samples, *An. minimus* had shorter knockdown times than *An. dirus*. These results indicate that permethrin on the uniforms remained active after 12 weeks of regular field use. In terms of mosquito mortality, we found that uniform samples had an average of 60% mortality for *An. dirus* and 95% for *An. minimus*, compared to 0% mortality for both mosquito species in the untreated uniform samples. These results show that permethrin-treated uniforms retained a significant quantity of permethrin that was still active in terms of mosquito knockdown and mortality after 12 weeks of regular use and washing in the field.

## 4. Discussion

We conducted a 12-week study with the LPA where we evaluated the usability, durability, and effectiveness of two vector control measures, permethrin-treated uniforms and metofluthrin spatial repellent, in a military field setting. In addition to collecting survey data to collect subjective data on usability and effectiveness, this is one of the first studies to measure the effectiveness of these interventions using a serological test for mosquito bite exposure. Furthermore, we used laboratory tests, including mass spectrometry and bioassays, to measure the concentration and activity of permethrin in the treated uniforms after 12 weeks of regular use and washing.

In this study, we found that, based on self-reported measures of mosquito presence and bite frequency, both metofluthrin use and permethrin-treated uniforms significantly reduced mosquito bites, with 86% of metofluthrin users and 73% of subjects that were given permethrin-treated uniforms reporting ‘much fewer’ bites. Based on serological responses to the *An. dirus* SG6 antigen as a measure of mosquito exposure, we found that permethrin-treated uniforms, but not metofluthrin, were associated with significantly lower (*p* < 0.05) anti-SG6 antibody responses after 12 weeks in the field. The discordance between the survey results and the serology testing could be attributed to differences in perceptions of protection as a result of the spatial repellent. Since metofluthrin works by repellency while permethrin requires tarsal contact, it is possible that metofluthrin resulted in the perception of fewer mosquitos being around the user and fewer mosquito bites. Permethrin, by contrast, would not reduce the number of mosquitos around the user, even if it did decrease the number of mosquito bites, which may affect its perceived efficacy. The lack of significant changes in antibody responses to *An. minimus* and *An. maculatus* SG6 antigens due to the vector control measures could be because these two species have been found to be markedly less exophagic and anthropophagic than *An. dirus* in Laos [19], and thus potentially less affected by interventions in a field environment.

Based on survey results, we found that both interventions were generally well tolerated, with over 75% of subjects reporting no major side effects or discomforts. However, we did find that approximately 11% of subjects that were given metofluthrin reported complaints of ‘foul smell’, while 14% of subjects that were given permethrin-treated uniforms reported complaints of ‘itchiness’, indicating room for improvement in the development of future spatial repellents and insecticides. Finally, using laboratory analyses, we showed that even after 12 weeks in the field with regular washing and use, the permethrin-treated uniforms maintained a significant concentration of permethrin that remained biologically active and induced knockdown and mortality in *Anopheles* mosquitos in a laboratory bioassay, demonstrating the durability of permethrin treatment in uniforms used in field settings.

Recent field evidence indicates that permethrin-treated uniforms provide protection against mosquito bites and malaria transmission in operational settings. A trial along the Cambodian–Thai border demonstrated reductions in *Plasmodium vivax* parasitemia among personnel wearing treated uniforms compared to controls [20]. These findings align with bioassay and field studies showing that impregnated clothing can reduce mosquito landing and biting rates [21], although efficacy may decline with washing, wear, and insecticide resistance [22,23,24]. Overall, permethrin-treated clothing represents a practical complement to standard vector control measures, particularly for mobile populations and outdoor workers in endemic regions.

There were several limitations to this investigation. First, the study interventions were not blinded and it is possible that knowledge of what vector control measures they were provided would have influenced the subjective assessments of the volunteers about the effectiveness in terms of mosquito presence and biting frequency. Second, because of logistical constraints, individual military units or cohorts were given the same intervention, rather than randomizing individuals within each unit to receive particular vector control measures. As such, any cohort-level differences in terms of vector exposure could have confounded the results seen here. Third, due to resource constraints, retrospective laboratory analysis of uniform samples was restricted to a handful of representative treated and untreated uniform samples. Fourth, compliance in terms of use of the vector control measures (spatial repellent or treated uniforms) was not strictly monitored during the 12-week field exercise, and it is possible that compliance varied from unit to unit, which could affect the results. Fifth, permethrin-treated uniform conditions in this study included provision of DEET repellent because this is the standard vector control measure used by the LPA. It is possible some of the effect of permethrin in this study was confounded by the effect of DEET, but this risk was mitigated by the fact that DEET-based repellents are widely available in Laos, and as such its use was likely varied but widespread throughout the study population regardless of study condition. Finally, a relatively small number of treated (*n* = 3) and untreated uniforms (*n* = 2) was used to assess permethrin treatment durability, which could limit the interpretability of the findings, but the bioassay efficacy results were largely consistent across the samples tested.

## 5. Conclusions

In this multi-arm unblinded study of the use of metofluthrin spatial repellent and permethrin-treated uniforms in the LPA in a 12-week field exercise, we found that both interventions were generally well received by subjects who reported that the vector control measures resulted in significantly lower mosquito presence and fewer mosquito bites. We found that both interventions were generally well tolerated but that 10–15% of participants complained about foul smell or itchiness. Using a serology test for mosquito bite exposure, we found that permethrin-treated uniforms were associated with a significantly reduced antibody response to the *Anopheles* salivary antigen SG6 after 12 weeks of the field exercise. Finally, we found that the permethrin in the treated uniforms was durable, with significant concentrations found in the uniform fabric after 12 weeks of regular field use and wear and that this concentration was bioactive with respect to *Anopheles* mortality in a laboratory bioassay. Overall, our findings support the general use of permethrin-treated uniforms and/or metofluthrin spatial repellent in military field settings and highlight areas for improvement in vector control measures for military units operating in regions or environments that are endemic for mosquito-borne infectious diseases such as malaria.

## Data Availability

The dataset supporting the conclusions of this article is included within the article and its additional files.
